# A Deep Learning Approach to Visualize Aortic Aneurysm Morphology Without the Use of Intravenous Contrast Agents

**DOI:** 10.1097/SLA.0000000000004835

**Published:** 2023-01-10

**Authors:** Anirudh Chandrashekar, Ashok Handa, Pierfrancesco Lapolla, Natesh Shivakumar, Raman Uberoi, Vicente Grau, Regent Lee

**Affiliations:** * Nuffield Department of Surgical Sciences, University of Oxford, Oxford, United Kingdom; † Department of Vascular Surgery, Oxford University, Hospitals, NHS Foundation Trust, United Kingdom; ‡ Department of Radiology, Oxford University Hospitals, NHS Foundation Trust, United, Kingdom; § Department of Engineering Science, University of Oxford, Oxford, United Kingdom

**Keywords:** aortic aneurysms, computer vision, computerized tomography, contrast-enhanced computerized tomography, CTangiography, deep learning, generative adversarial network

## Abstract

Intravenous contrast agents are routinely used in CT imaging to enable the visualization of intravascular pathology, such as with abdominal aortic aneurysms. However, the injection is contraindicated in patients with iodine allergy and is associated with renal complications.

**Objectives:**

In this study, we investigate if the raw data acquired from a noncontrast CT image contains sufficient information to differentiate blood and other soft tissue components. A deep learning pipeline underpinned by generative adversarial networks was developed to simulate contrast enhanced CTA images using noncontrast CTs.

**Methods and Results:**

Two generative models (cycle- and conditional) are trained with paired noncontrast and contrast enhanced CTs from seventy-five patients (total of 11,243 pairs of images) with abdominal aortic aneurysms in a 3-fold cross-validation approach with a training/testing split of 50:25 patients. Subsequently, models were evaluated on an independent validation cohort of 200 patients (total of 29,468 pairs of images). Both deep learning generative models are able to perform this image transformation task with the Cycle-generative adversarial network (GAN) model outperforming the Conditional-GAN model as measured by aneurysm lumen segmentation accuracy (Cycle-GAN: 86.1% ± 12.2% vs Con-GAN: 85.7% ± 10.4%) and thrombus spatial morphology classification accuracy (Cycle-GAN: 93.5% vs Con-GAN: 85.7%).

**Conclusion:**

This pipeline implements deep learning methods to generate CTAs from noncontrast images, without the need of contrast injection, that bear strong concordance to the ground truth and enable the assessment of

important clinical metrics. Our pipeline is poised to disrupt clinical pathways requiring intravenous contrast.

In a computed tomography (CT) image, density variations across tissues translate to differences in tissue attenuation and subsequent radiodensities (measured in Hounsfield Units, HU).^1^ HU values are generally displayed as a greyscale, with brighter regions corresponding to higher attenuation (eg, bone and calcification) and conversely for darker regions (eg, fat and air). Where treatment of an artery is being considered, a detailed view of the arterial anatomy is required. In the example of abdominal aortic aneurysms (AAA, abnormal ballooning of the abdominal aorta), an intra-luminal thrombus (ILT) adherent to the aortic wall within the enlarging aneurysmal sac is present in 95% of cases^2^ (Supplementary Figure S1, http://links. lww.com/SLA/D12). Given the similarities in density, these regions cannot be readily distinguished using a conventional noncontrast CT (NCCT) image.

Clear visualization of these regions can only be achieved by injecting an intravenous (IV) iodinated contrast agent in a CT angiogram (CTA). IV contrast increases the luminal density and attenuation to distinguish the vascular tree from surrounding soft tissues.^1,3^ However, CTAs are associated with several disadvantages.^3,4^ CTAs are contraindicated in patients with iodine allergies, as most contrast agents are iodine-based. Complications include inadvertent arterial puncture by needle, and contrast leak causing skin irritation/damage.^4^ Additionally, contrast agents are nephrotoxic and have up to 12% incidence of reported acute kidney injury following use.^4^ This is especially a problem within the elderly population, who either have decreased baseline renal function or concomitant chronic kidney disease. There is a recognized risk of complete kidney failure in these patients, leading to renal dialysis and mortality.

We hypothesize that the raw data acquired from a NCCT can be used to differentiate blood and other soft tissues. Blood is predominantly fluid, with red/white blood cells whereas the adjacent ILT is predominantly fibrinous and collagenous, with red cells/ platelets. These individual components vary in ultrastructure and physical density, which should reflect in different (albeit subtle) HUs on a CT scan (either in individual HU values or in their spatial distribution/“texture”). We further hypothesized that using deep learning (DL) generative methods, these subtle differences can be amplified to enable simulation of contrast-enhanced images without the use of contrast agents.

In this study, we investigate the ability of generative adversarial networks (GANs) for this noncontrast to contrast image transformation task. These networks are a class of DL architectures whereby 2 neural networks train simultaneously, with 1 network focused on data generation (generator) and the other focused on data discrimination (discriminator). Designed to mimic how the human brain operates, these neural networks are sets of algorithms that attempt to understand the underlying relationships in the provided training data for a particular task. In this instance, these networks compete against each other to better learn the underlying statistical distribution of the training data. This allows for the generation of new examples from the same distribution.^5^ Many variations of the original GAN have been developed, including conditional GANs (Con-GANs1Conditional GANS are usually abbreviated as CGANs in the machine learning literature. Here, Con-GAN is used to avoid confusion with Cycle-GAN.) and cycle-GANs. The former learns the transformation between 2 paired distributions using a pixel-to-pixel approach.^6^ On the other hand, the Cycle-GAN is able to learn transformations between 2 distributions without the need for direct pairings between samples.^5^ Here, we assessed the performance of these DL generative models using 2 technical metrics [root-mean-square-error (RMSE), Sørensen-Dice score (DICE)] and 4 clinically important metrics (1. diameter, 2. cross-sectional area, 3. volume of the lumen and outer wall structure, and 4. AAA thrombus morphology).

## Methods

### Curation of CT Images From a Clinical Cohort

CT scans were acquired through the Oxford Abdominal Aortic Aneurysm (OxAAA) study. The study received full regulatory and ethics approval from both Oxford University and Oxford University Hospitals National Health Services Foundation Trust (Ethics Ref 13/ SC/0250). The OxAAA study was designed to investigate novel biomarkers in the context of AAA disease. Details regarding the OxAAA study have been previously published.^7^ The study complies with the principles outlined in the Declaration of Helsinki. Each patient gave consent for the use of clinical and imaging data for research analyses. This research project (contents of this manuscript) was inspired by our attempt to utilize historic CT scans acquired during the AAA surveillance period to discover novel signatures of AAA growth,^8^ as many of these historic CT scans were noncontrast scans.

As part of the routine preoperative assessment for aortic aneurysmal disease, a NCCT of the chest/abdomen/pelvis and an arterial phase CTA was performed. CTA images were obtained in helical mode with a predefined slice thickness of 1.25 mm. On the other hand, NCCT images were obtained with a predefined slice thickness of 2.5 mm. Paired contrast and NCCT were anonymized before subsequent analysis. Seventy-five (75) patients with paired NCCT and CTA images (11,243 pairs of images) were randomly selected from the OxAAA cohort and were used for model training. An independent set of 200 patients with paired NCCT and CTA images (29,468 pairs of images) were selected and served as a validation cohort. Paired images were segmented using our proprietary automated DL pipeline^9^ and registered/shifted to ensure aortic overlap for subsequent analysis (Supplemental Methods, http://links. lww.com/SLA/D12).

### EXPERIMENT 1: HU Sampling Between Different Regions in the AAA

**Figure 1:**
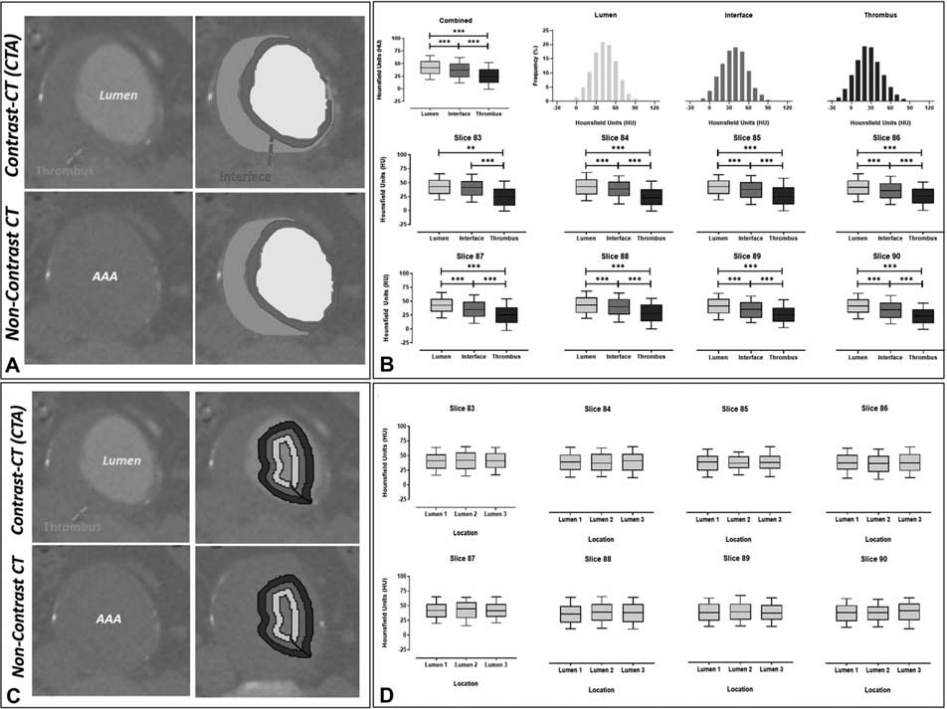
Axial slices from both the contrast and noncontrast CT scans. A, Demarcated regions display the thrombus (blue), the lumen (yellow), and the interface between the regions (red). B, Hounsfield unit sampling of the lumen, interface, and thrombus with histograms displaying the frequency of HUs within each region. Analysis at each axial slice was performed and showed differences in HU intensity between the lumen, interface, and thrombus regions. C, Concentric sampling within the lumen as demarcated by the pink, magenta, and purple, was used as a negative control for this experiment. D, Hounsfield unit sampling of the lumen at multiple locations indicated a minimal difference in HU intensity. CT indicates computed tomography;HU, Hounsfield Unit.

To investigate the regional differences within an aneurysm, 100 paired axial slices were selected from 10 random patients. These slices were visually validated for registration accuracy by 2 blinded reviewers (NS and PL) and were sampled for the underlying HU distribution at the lumen, ILT, and interface locations (Fig. 1). These visually indistinct regions on the NCCT images were identified from their paired CTA images. To account for slight discrepancies in the image registration process, and minimize sampling errors, the thrombus (blue) and lumen (yellow) areas were shrunk by 20% at the adjoining border. The zone between the 2 regions was demarcated as the interface (red). This delineation is clearly indicated in Figure 1. The average HU intensities within each region were compared using a One-way-ANOVA. As a negative control, concentric sampling within the blood lumen in each of the NCCT images was performed.

### EXPERIMENT 2: GANs: Cycle-GAN and Con-GAN

In this study, the cycle-GAN and the Con-GAN were used for the noncontrast to contrast image transformation task (Supplementary Figure S2, http://links.lww.com/SLA/D12). Model architectures and training details are described in the Supplemental Methods, http://links.lww.com/SLA/D12.

### GAN Models Training and Evaluation

A 3-fold cross-validation paradigm with a training/test data split of 50:25 patients (~7500: ~3750 2D axial slices) was employed. Model performances were evaluated using 2 metrics: RMSE and Sorensen-DICE score. The former is a commonly used metric in image transformation tasks to assess the pixel-by-pixel difference between the simulated CTA image and the ground truth (GT) (ie, paired CTA image). The latter is a quantitative similarity assessment of regions within sets of images.^10^ Here, DICE_I_ was calculated to assess overlap in the inner lumen between the simulated pseudo-contrast and GT CTA images. Additionally, DICE_C_ measures aortic shape overlap between the simulated and GT images. This is a surrogate measure to evaluate both registration accuracy and shape alterations induced by the generative network.

### CT Image Quality and GAN Model Performance

The NCCT images used in this study were acquired using a variety of settings (eg, x-ray tube current, rotation time, and pitch factor) that may impact image quality. The combination of x-ray tube current and rotation time determines the amount of incident x-ray photons, which is directly proportional to image quality. Given that these DL platforms utilize these images and the underlying pixel relationships as the basis for transformation, we further hypothesized that NCCT images obtained with greater x-ray tube currents would result in qualitatively and quantitatively better simulated CTA images. This was evaluated by comparing the patients with best and worst image transformations to define an optimal cut-off criterion of image quality and for subsequent analysis.

### EXPERIMENT 3: Evaluation of Clinically Important Metrics Using Simulated Pseudo-contrast Images

The following metrics were obtained from the GT CTA and simulated pseudo-contrast images using an in-house algorithm in MATLAB^11^: 1-D measurements: maximum lumen and aortic wall diameters; 2-D measurements: blood lumen and ILT areas; 3-D measurements: blood lumen and ILT volumes. Bland Altman plots and correlation coefficient analysis were performed for each to assess bias and the strength of association between the output of the GAN models and the GTs. Bias measurements are reported along with its 95% confidence interval. In addition, the spatial morphology of the ILT was extracted using an in-house algorithm in MATLAB. This is an important feature that is used to plan surgical intervention and can influence postsurgical outcomes.^7^ Based on our previous work, spatial morphology of the ILT can be categorized into 7 categories (Supplementary Figure S3, http://links.lww.com/SLA/D12) as previously detailed.^7^

### EXPERIMENT 4: Evaluation of GAN Models on the Independent Validation Cohort

An independent set of 200 patients with paired NCCT and CTA images (total of 29,468 pairs of images) were selected from the OxAAA study and served as a validation cohort. The con- and cycle-GAN models from each validation fold (3) were used to generate pseudo-contrast images of the aorta/aneurysm. The outputs of each model were averaged to generate the final pseudo-contrast images. As a result, for each NCCT image, 2 pseudo-contrast images were generated: 1 from the averaged Con-GAN outputs and the other from the averaged Cycle-GAN outputs. Following image transformation, CTA images were provided to assess transformation accuracy. This was completed using the same methods and metrics as described above. Subsequently, the effect of aneurysmal size (as measured by maximum AAA diameter) and aneurysm shape (fusiform vs saccular) on GAN performance was investigated. The aneurysmal shape was quantified using the non-fusiform index (NFI), which is a 3-D shape index that describes the deviation of the aneurysmal sac from an ideal fusiform shape. This metric was derived from Martufi et al^12^ and is explained in detail within the Supplement, http://links. lww.com/SLA/D12.

### EXPERIMENT 5: GAN Model Training and Evaluation for the Simulation of Aortic Side Branches

From Experiment 4, we selected the GAN model with superior performance in simulating aortic/aneurysmal-specific features for subsequent analyses. The training data for this experiment consisted of 2-D axial slices centered around the aorta/AAA with surrounding tissue present (aortic region-of-interest, ROI; Supplementary Figure S4, http://links.lww.com/SLA/D12). As a result, this generative network was trained to concurrently simulate both intra- (aortic lumen, thrombus morphology) and extra-aortic/AAA features (main aortic side branch origins: Coeliac artery, superior mesenteric artery, renal arteries, and iliac arteries). Same model training (3-fold cross-validation paradigm, n = 75 patients) and evaluation parameters were utilized for this experiment. Maximum aortic side branch diameter was manually obtained in a blinded fashion from both the contrast-enhanced and the GAN-generated pseudo-contrast CT images for the 6 major branches originating from the descending/ abdominal aorta, wherever available (1. Coeliac artery, 2. Superior Mesenteric Artery, 3–4. Left/Right Renal Arteries, and 5/6. Left/ Right Iliac Arteries). Mean average error between measurements were reported for each branch. Student t-testing was performed to assess for statistical significance.

## Results

### HU Intensities Differ Between Regions of a AAA in NCCT Images

There are discernable differences between the HU frequency distributions of each region (Fig. 1B). Average HU intensity in the NCCT images differed significantly between all 3 regions (blood lumen vs thrombus, blood lumen vs interface, and interface vs thrombus) for all patients assessed. Figure 1B demonstrates the significant differences in HU intensities between the thrombus and lumen for 8 axial slices obtained from 1 patient. Furthermore, the blood lumen/thrombus interface also differed significantly from the other 2 regions, indicating a gradual change from lumen to the thrombus. As a comparison, no significant HU differences were observed following concentric sampling within the lumen (Fig. 1C and D).

### Generative Models Can Simulate Contrast Images Using Noncontrast Images

**Figure 2:**
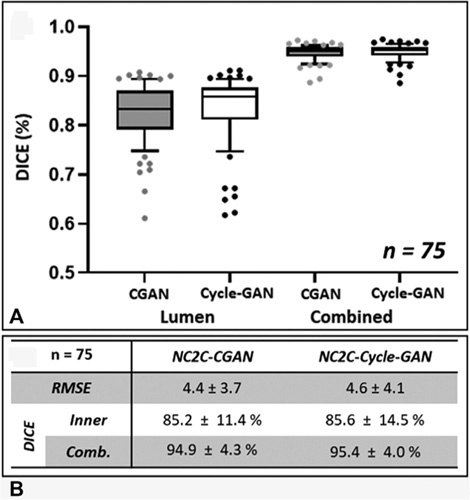
Transformation accuracy within the testing cohort. A, Box plots of averaged DICE scores per patient within the testing cohort for the lumen (DICEI) and the combined aortic mask (DICE_C_) segmentations. Segmentations are derived from model (Cycle-GAN/Con-GAN) predictions. Individual data points outside the 10-90th percentile are highlighted. B, RMSE/DICE scores of generated pseudo-contrast axial images. DICE indicates Sørensen-Dice score; RMSE, root-mean-square-error.

During model training, the RMSE between the simulated pseudo-contrast images and GT (CTA) images decreased with each epoch (training cycle) to plateau at 3.8 ± 0.8 and 3.9 ± 0.6 for the con-GAN and cycle-GAN, respectively. Similarly, the DICE_I_ increases with epoch duration to plateau at 91.8% ± 0.6% and 92.0% ± 0.4% for the con-GAN and cycle-GAN, respectively. Figure 2 indicates the RMSE and DICE scores for the images generated from the testing cohorts across the 3 folds using both GANs (n = 11,243 images, 75 patients). A per-patient transformation accuracy was derived by grouping the 2D-axial images and their respective DICE scores by the patient. With regards to DICE_I_, the median performance of the cycle-GAN is greater than that of the Con-GAN. Of note, there were multiple overlaps between the outliers below the 10th percentile of cycle-GAN and Con-GAN (6/7) networks. This suggests that there may be image properties inherent to this subgroup of NCCTs leading to decreased transformation accuracy.

### CT Image Quality Affects NCCT Transformation Accuracy

Comparing the image properties of the NCCT scans below the 10th percentile and above the 90th percentile highlighted 1 key difference with regards to the x-ray tube current (I_tube_) used during image acquisition (Supplementary Figure S5, http://links.lww.com/SLA/D12). Scans obtained with lower I_tube_ values produced images with poor transformation accuracy. Subsequently, a threshold criterion of above the 15th percentile of I_tube_was implemented. This equated to a selection criterion of images obtained with Itube > 80 mA. Apart from tube current, there were no observable differences between other CT acquisition parameters (mean ± SD) for the 2 subsets of cases. Notably, no difference was observed in spiral pitch factor (excluded: 1.08 ± 0.17, included: 1.10 ± 0.18, *P* = 0.42), slice thickness (excluded: 2.5 mm, included 2.5 mm, *P* = 1.0), and total collimation width (excluded: 35.9 ± 8.2, included: 34.2 ± 9.3, *P* = 0.33).

**Figure 3:**
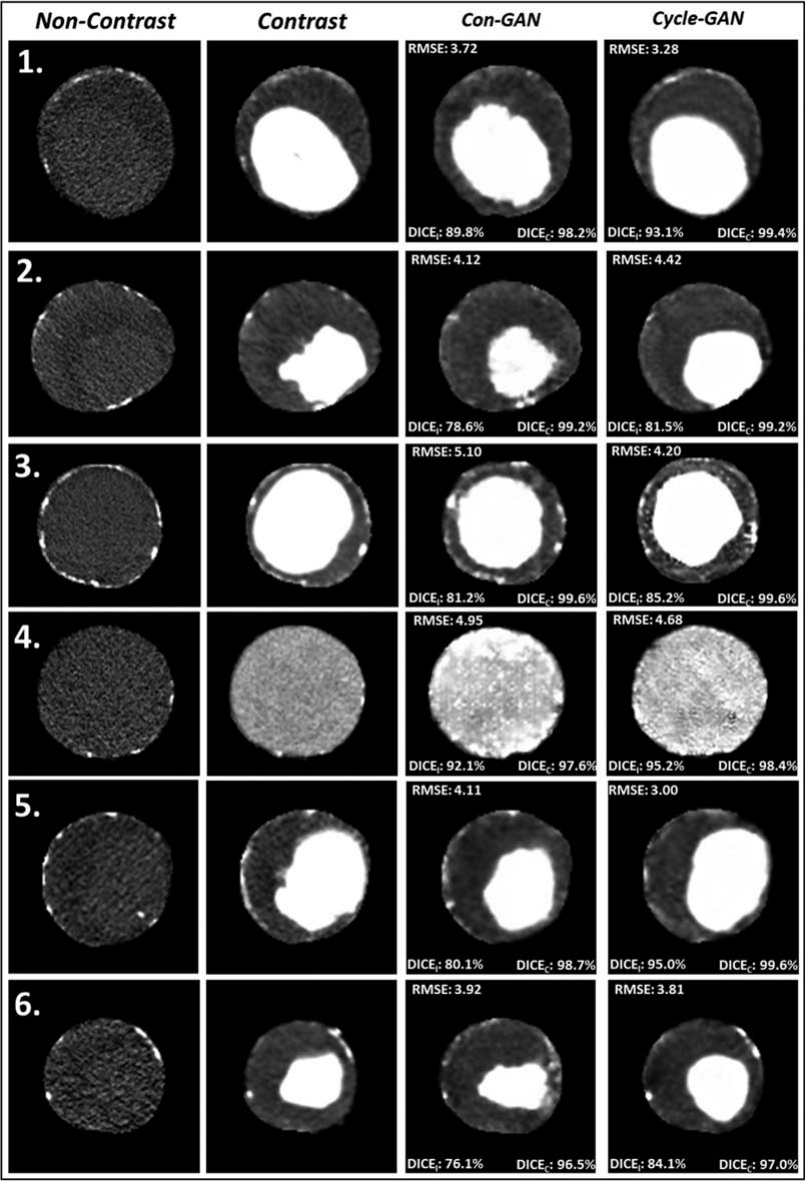
Pseudo-contrast images generated using the Con-GAN and Cycle-GAN architectures from 6 different patients alongside their respective NCCTand CTA axial slices. Using the CTA axial images as GT, RMSE, and DICE scores for the inner lumen (DICE_I_) and combined aorta (DICE_c_) are displayed. CTA indicates CT angiogram;DICE, Sørensen-Dice score; GAN, generative adversarial network;GT, ground truth; NCCT, noncontrast CT; RMSE, root-mean-square-error.

**Figure 4:**
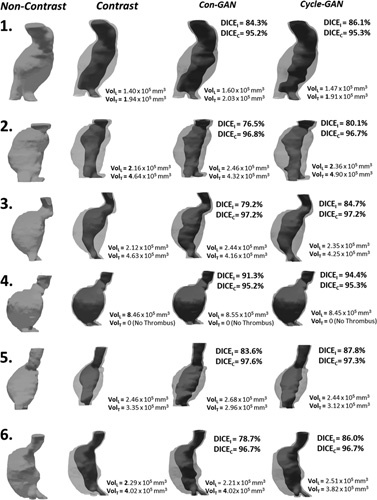
Segmented volumes generated from the Con-GAN and Cycle-GAN architectures. Volumes from 6 patients, the same as seen in Figure 6, are displayed alongside their respective NCCT and CTA volumes. DICE_I_, DICE_c_ and lumen/thrombus volumes are calculated and displayed for each aneurysmal region. CTA indicates CTangiogram; DICE, S0rensen-Dice score; GAN, generative adversarial network; NCCT, noncontrast CT.

Exclusion of these 12 patients (I_tube_ < 80 mA), resulted in improvements in RMSE, and in DICE_I_ for both generative models (Supplementary Figure S6A and B, http://links.lww.com/SLA/D12). DICE_C_ scores remained unaffected by this exclusion. The blood lumen generated from the Cycle-GAN bears a closer resemblance to the GT image as compared to that generated by the Con-GAN, as reflected by the superior DICE_I_ scores. On the other hand, DICE_C_ scores were identical for both sets of model predictions. This is apparent in Figure 3, which illustrates paired CTA/NCCT axial slices alongside their pseudo-contrast images from 6 different patients. The aneurysm volume for each of these patients is illustrated in Figure 4. Correspondingly, the inner lumen areas and thrombus volumes derived from the cycle-GAN model outputs are better approximations to those derived from the GT compared to that of the con-GAN.

### Cycle-GAN Outperforms Con-GAN in Simulating Contrast CT Images Based on the Measured Clinical Metrics

Evaluation of aneurysm morphology is useful in defining the biological behavior of an AAA during the natural history of the disease.^7,13–16^ Measurements derived from the 2 GAN models' outputs were compared against those obtained from the GT.

### 1-D Measurements

**Figure 5:**
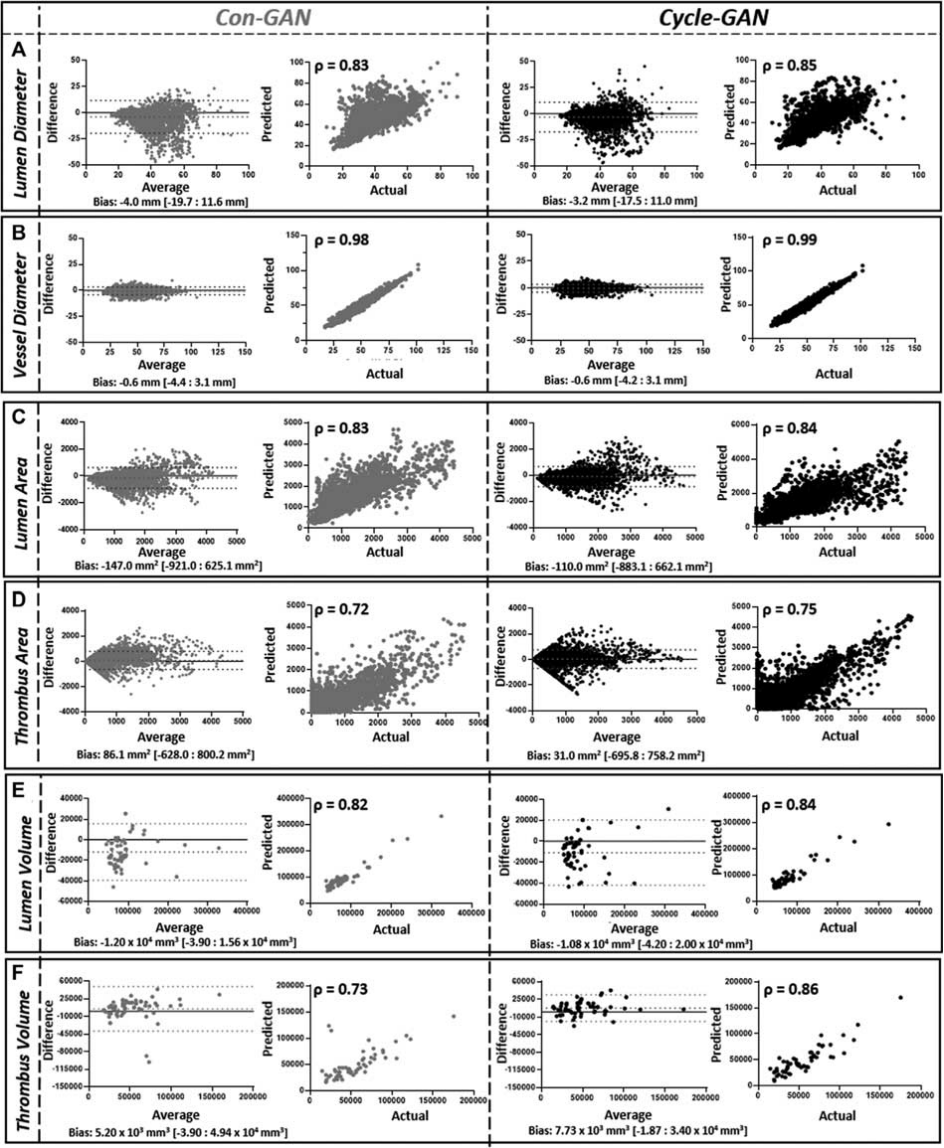
One-dimensional diameter (A and B), 2-dimensional area (C and D), and 3-dimensional volume assessment (E and F) of generated images. Bland-Altman plots and correlation-coefficient analysis comparing the measurements of generated images compared against those derived from ground truth segmentations. Measurements derived from Con-GAN and Cycle-GAN outputs are illustrated in grey and black, respectively. Spearman correlation coefficients (*p*) are indicated on the graphs *(P **<*** 0.01 for all comparisons). GAN indicates generative adversarial network.

The Cycle-GAN model is better at approximating the maximum lumen diameter than the Con-GAN model (Fig. 5A). On the other hand, both models have similar strengths in determining the outer vessel wall diameter (Fig. 5B). Maximum inner lumen and outer vessel wall diameters extracted from the model outputs correlated strongly with GT measurements (Cycle-GAN, ρ = 0.85 and 0.99, *P <* 0.01; Con-GAN, ρ = 0.83 and 0.98, *P <* 0.01).

### 2-D Measurements

The Cycle-GAN model again performed better than the Con-GAN model (Fig. 5C and D). Thrombus area in each axial slice as determined by the Cycle-GAN and Con-GAN models is on average 9.3% ± 11.5% and 9.4% ± 12.2% different from GT measurements.

### 3-D Measurements

The Cycle-GAN better approximates the 3D-lumen and thrombus volume measurements than the Con-GAN (Fig. 5E and F). Lumen volumes derived from both models (Cycle-GAN: ρ = 0.84, *P <* 0.01; Con-GAN: ρ = 0.82, *P <* 0.01) and thrombus volumes (ρ = 0.86, *P <* 0.01) from the Cycle-GAN correlated strongly with the manually derived measurements.

**Figure 6:**
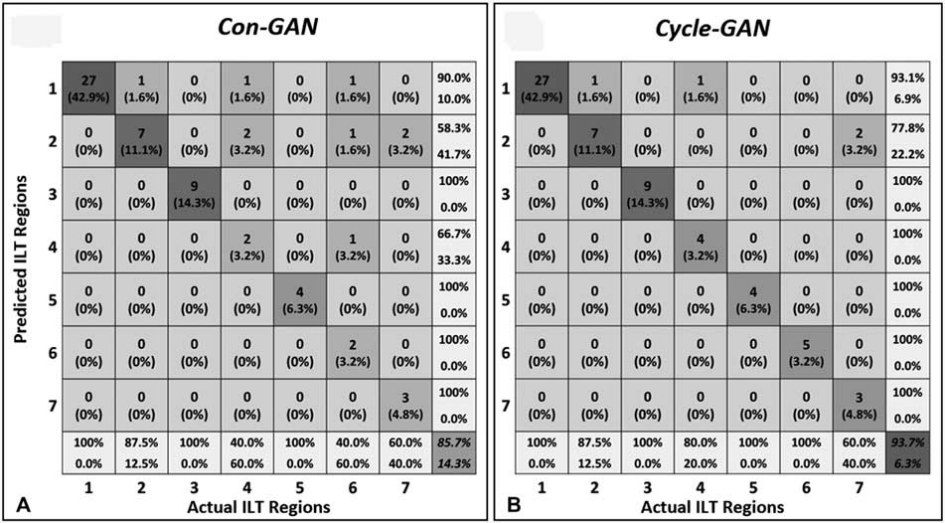
Confusion matrices comparing ILT regional classifications between generated images and ground truth segmentations. Segmentations derived from model outputs (Con-GAN (A), and Cycle-GAN (B)) were classified into 7 categories as per Fig. S3 and evaluated against that determined by the ground truth segmentations. GAN indicates generative adversarial network; ILT, intra-luminal thrombus.

Pseudo-contrast images within the aneurysmal region produced by Cycle-GAN (Fig. 6A) had an ILT classification accuracy of 93.5%, which outperforms that produced by the generative images of the Con-GAN model (85.7%, Fig. 6B). Examples of the discrepancies between the models are illustrated in Supplementary Figures S7 and S8, http://links.lww.com/SLA/D12.

### Similar GAN Performance is Observed Within the Independent Validation Cohort

Of the 200 independent cases, 35 were subsequently excluded as they were obtained at tube currents <80mA (n = 25) or at unknown tube currents (n = 10). RMSE between the generated pseudo-contrast and CTA images for the 165 patients (Cycle-GAN: 4.2 ± 3.8 and Con-GAN: 4.2 ± 3.7) were similar to that observed in the training set. DICE accuracy of the inner lumen again showed superior performance of the cycle-GAN (84.1% ± 7.2%) when compared against that of the Con-GAN (83.2% ± 7.7%). Extracted 2- and 3-D measurements from this refined testing cohort further supported the finding that the Cycle-GAN is superior at simulating CTA images of the AAA.

As a post-hoc analysis, we compared the GAN performance between the excluded cohort (n = 35) and the rest (n = 165). Area and volume measurements of the AAA lumen/thrombus derived from NCCT images obtained at >80mA were closer to measurements derived from GT annotations than the excluded cohort (*P* < 0.001 for all comparisons, Supplementary Table S1A and B, http:// links.lww.com/SLA/D12). Additionally, pseudo-contrast images within the aneurysmal region produced by Cycle-GAN had an ILT classification accuracy of 90.6%, which outperforms the one produced by the generative images of the Con-GAN model (83.8%). This further supports the use of tube currents as a criterion to assess the quality of DICOM data for analysis.

The role of aneurysmal size (maximum AAA diameter) and shape (NFI) was compared against GAN performance for both models. Supplementary Figure S9A–D, http://links.lww.com/SLA/ D12 illustrates 4 aneurysms alongside their respective “ideal” fusiform shape. Aneurysms with a predominantly fusiform shape had lower NFIs compared to those aneurysms with a predominantly saccular shape. This supports the classification of aneurysm shape using NFI. Both AAA size and NFI had no significant impact on transformation accuracy as assessed by DICE score of the inner lumen from both the Cycle- and Con- GANs (Supplementary Figure S9E, http://links.lww.com/SLA/D12). This suggests that trained generative networks do not have an implicit AAA size or shape preference.

### Cycle-GAN Can Simulate Extra-aortic/AAA Features Including Aortic Side Branches

**Figure 7:**
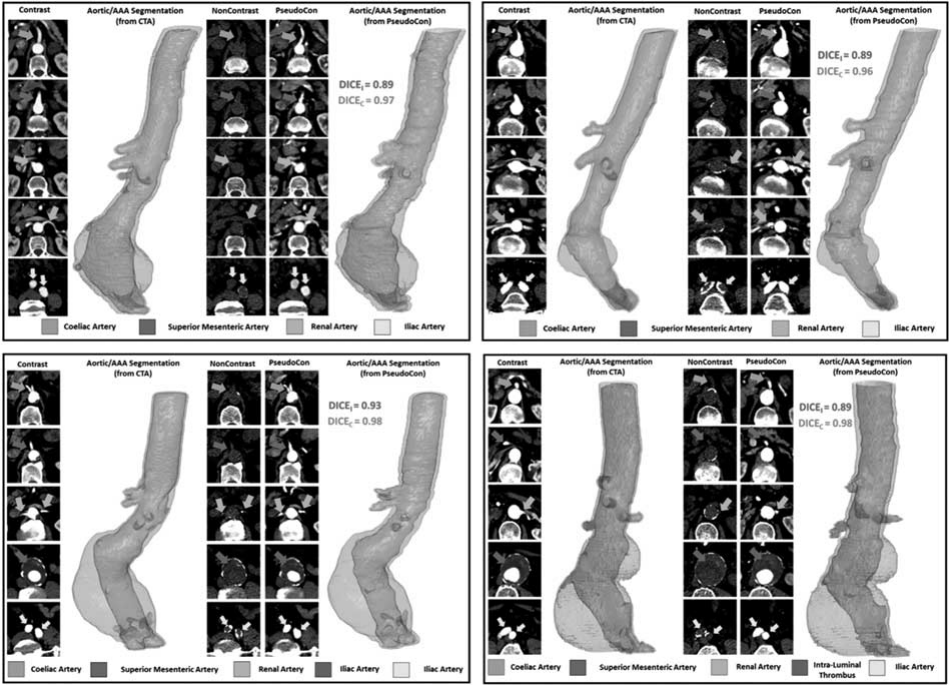
Pseudo-contrast CT images are displayed alongside their respective contrast and noncontrast CT Images for 4 patients. Arrows indicate the branch arteries (celiac artery, superior mesenteric artery, renal arteries, and iliac arteries) and the intraluminal thrombus are highlighted wherever visible. Aortic segmentations including the side branches are generated from both the CTA (gold standard) and the pseudo-contrast CT and DICE scores comparing the segmentations are displayed. CTA indicates CT angiogram; DlCE, Sørensen-Dice score.

Given thatthe Cycle-GAN generated superiorresults against that of the Con-GAN, we traineda Cycle-GAN to identifyan expanded ROI surrounding the aorta. This concurrently simulates both intra- (aortic lumen, thrombus morphology) and extra-aortic/ AAA features (major abdominal aortic side branches). Despite the expanded input data size (ie, more information to “learn” from), its ability to extract intra-aortic/aneurysmal features are comparable to the previously trained models (smaller ROI/less information to “learn” from) (Supplementary Table S1C, http:// links.lww.com/SLA/D12). Figure 7 illustrates generated pseudocontrast images alongside their respective CTA and NCCT images for 4 patients within the validation cohort. Branch arteries arising from the aorta are visible in all patients. Supplementary Table S2, http://links.lww.com/SLA/D12 highlights the maximum diameters for each branch, obtained in a blinded fashion, from pseudocontrast CT and its corresponding CTA. There is no difference between the diameter for all 6 major aortic side branches measured using the CTA (GT) or the pseudo-contrast CT (derived from the NCCT images).

## Discussion

The primary objective of this study was to investigate whether there are subtle differences between visually indistinguishable regions within the NCCT image. This was required to ensure that the images contained the necessary information for the DL method to generate anatomically-correct visualizations. This was achieved by comparing the HU intensity distributions between regions in the ILT-filled aneurysm. Visually, axial slices within the AAA seem uniform on the NCCT image and the histograms for each of the regions (lumen, interface, and thrombus) display considerable overlap. However, average HU intensity was significantly different between all 3 regions for all patients assessed and a gradual HU change was observed from the aortic lumen to the ILT. This highlights that there are differences, albeit subtle, between the regions that can be exploited and enhanced to estimate the CTA image.

In addition to average HU intensity, the differences between these visually in-distinct regions can be captured using multiple firstorder radiomic features (eg, uniformity, kurtosis, and skewness -Supplementary Figure S10, http://links.lww.com/SLA/D12). Radiomics employs advanced data-characterization algorithms to extract underlying pixel relationships and has been used to uncover potential disease features that fail to be appreciated visually. Here, these significant radiomic differences between lumen, thrombus, and its interface in NCCT images strongly support the validity of this image transformation task. The generative networks likely utilize this higher-order information to enhance its performance.

The study's secondary objective was to investigate if DL models (Cycle- and Con-GAN) could extract the subtle differences between soft tissue components in NCCT images within the context of AAA disease and generate CTA images. For both GANs, a 3-fold cross-validation approach was employed during training. There was no data leakage between cohorts and the testing cohort for each fold contained a unique set of patients. The 2-D training/testing data was obtained by first isolating the aorta, axial sub-sampling within the volume, and centering the aorta within the extracted 2-D slice. Isolating the aorta by removing surrounding organs and tissues reduces the amount of noise presented to the model. Additionally, centering the aorta in each axial 2D slice reduces the spatial variation of the aorta seen by the generative models. This theoretically should maximize the information learned by the GAN networks for the NCCT to CTA image transformation task. RMSE, a quantitative measure of image difference, and DICE scores for the inner lumen and combined aortic mask were used to optimize the training parameters. DICE accuracy of the inner lumen is an appropriate measure of transformation accuracy as it evaluates the primary goal of the generative models.

It was apparent that CT image quality determines transformation accuracy. Exploring the DICE_I_ metric within the testing cohort highlighted certain patients with not only decreased transformation accuracies but also greater DICE_I_ variability within the aortic volume. Here, we hypothesized that poor quality NCCT images have correspondingly poor CTA reconstruction accuracies. Image quality and the extent of distortion (“signal to noise” ratio) of NCCT images is directly related to the number of x-ray photons incident within the target volume. Accordingly, the number of x-ray photons is directly related to the x-ray tube current (I_tube_), which is the rate of photon production within the x-ray tube, and CT rotation time. Given that CT rotation time is usually constant, we reasoned that images obtained with decreased I_tube_ have an increased likelihood of generating CTA images with decreased accuracy. Therefore, a stringent selection criterion was imposed to exclude potential outliers with I_tube_ below the 15th percentile (<80 mA). Future studies investigating the role of CT acquisition parameters (eg, slice thickness, kVP, tube current) for this transformation task in greater detail is required to determine the limitations of this technique.

An unexpected observation was that cycle-GAN outperformed the con-GAN. In the original context, we hypothesized that the Con-GAN would have superior performance to the Cycle-GAN as it is able to learn pixel-to-pixel transformations between image pairs. (This is not the case for the Cycle-GAN as data is not introduced as paired images.) This superiority was unexpected but can be rationalized to its underlying network architecture and the multiple training losses (eg, cycle-consistency and identity loss terms). This interesting observation sheds some unique philosophical insight into the nature of “learning” by neural network and its similarities of human learning for this particular task. For a cycle-GAN, the neural network learns heuristically from all images of the paired GT data, without being constrained to specific pixel relationships as that of a Con-GAN. It is possible, however, this observation is specific to the dataset utilized here.

We showed that these generative models enable the visualization of aortic aneurysm morphology in CT scans obtained without the use of IV contrast and that transformation accuracy is independent of AAA size or shape. Extracting diameter measurements is required in AAA management as it guides the frequency of aneurysm surveillance and determines the timing of surgery.^13,17^ 2-D cross-sectional area measurements of the aneurysm have been shown to complement the 1-D diameter measurements as diameter measurements can be subject to substantial interobserver variability and at times may fail to represent the 3-D growthofthe aneurysm. As a result, cross-sectional area measurements have been shown to have the lowest variability in assessing aneurysm size.^16^ Evolution of 3-D indices, especially thrombus volume, is linked to AAA progression, rupture risk, and even the incidence of adverse cardiovascular events.^15,18^ Assessing ILT spatial morphology is important for surgical planning and has been shown to influence postoperative outcomes. We have previously reported that the spatial morphology of native ILT correlates with the onset of type 2 endoleak, which is an adverse outcome following endovascular surgical repair of aneurysms.^7^ This is a task that is not possible from the original NCCT image and if achieved, reinforces the clinical impact of using generative networks for this image transformation task. For the first time, the results highlight the ability to assess the regional location of the ILT from NCCT image with high classification accuracy.

In addition, we showed that a Cycle-GAN trained on 135*135 mm (144 ×; 144 pixels) ROIs surrounding the aorta is able to robustly visualize not only aortic/AAA morphology but also extraaortic structures, including its side branches. The accuracy of the side branch visualization was assessed by measuring the maximum diameter of each branch from the descending/abdominal aorta. The results support the ability to capture this measurement with a mean average error of **~** 1.5 mm (1-2 pixels). Using GANs for pseudo-contrast CT visualization of the aorta/AAA, its ILT and its side branches from a NCCT is a novel technique and presents clinicians with a safer alternative to the routinely obtained contrast-enhanced CTA. Future studies are required to determine its clinical utility, such as using this alternative imaging method to plan for endovascular grafting.

This DL approach described here can also be applied to reconstruct other anatomical structures (veins, solid organs, etc) without the need for contrast administration. Beyond the potential clinical utility, our method can be applied for research analysis. There is a growing body of literature on the role of the intraluminal thrombus and AAA growth.^15,19,20^ The ability to characterize ILT using noncontrast-enhanced CT scans greatly expands the scope for research in this topic, without the need to subject research participants to contrast injection.

## Conclusions

We present a DL approach to visualize aortic aneurysm morphology and its side branches without the use of IV iodinated contrast agents. This platform technique can be applied to different anatomical structures for research and eventually clinical applications.

## Supplementary Material
