# Supplementary material for: A Deep Learning Approach to Visualize Aortic Aneurysm Morphology Without the Use of Intravenous Contrast Agents
Source: Ann Surg. 2023 Jan 10;277(2):e449–59. doi: 10.1097/SLA.0000000000004835 (PMC8691372; doi:10.1097/SLA.0000000000004835)
Supplement: Supplementary file 1 [file sla-277-e449-s001.docx]

**Supplemental Methods**

***Segmentation of CT Images (Training Dataset)***

Segmentation of the aortic inner lumen (in contrast-enhanced CTAs) and aortic outer wall (for both NCCT and contrast-enhanced CTAs) were performed using our proprietary automated deep learning segmentation pipeline^1^. This platform takes advantage of attention-gated U-Nets for both region-of-interest (ROI) selection and multi-label aortic segmentation^2^. The segmentations were visually assessed against the source DICOM images and any minor errors in segmentation were manually corrected using the open-source ITK snap software^3^.

The segmentation pipeline consists of two primary components: aortic region-of-interest (ROI) detection followed by aortic segmentation. The former used down-sampled resolution, isotropic CT images as input to isolate the location of the aorta. The isolated region within the higher resolution CT served as the input for the latter. From contrast-enhanced images, the attention-gated U-Net would simultaneously extract the lumen and outer wall structure labels as independent labels. On the other hand, the network would extract the entire aorta as a singular label from non-contrast CT images. This pipeline was trained and tested using a 3-fold cross-validation protocol to segment the aorta from the aortic root to the iliac bifurcation.

Overall segmentation accuracy for the thoracic and abdominal aorta in contrast-enhanced CT images were 95.0 ± 3.9 % and 93.7 ± 1.8 %, respectively. Similarly, segmentation accuracy for the abdominal aorta in non-contrast CT images was 93.5 ± 2.3 %. The accuracy metrics were comparable to those derived from inter-observer variability assessments. This pipeline allows for the extraction of complex morphological information from non-contrast images. This method serves as the foundation for all segmentations generated in this manuscript.

***Registration (alignment) of Contrast-Enhanced Scans to Non-Contrast Scans***

To account for voluntary and involuntary movement by the patient between scans, it was necessary to register (align) the contrast-enhanced images obtained to the non-contrast image plane. In order to optimize the registration of the aorta between these two images, the segmented volumes from the CTA and NCCT images were first registered using an non-rigid b-spline registration program in MATLAB^4^. In addition to the registration accuracy calculated within the program, the Sørensen–Dice (DICE) score was calculated to gauge the similarity of the moving/registered image (CTA) with the static image (NCCT). The DICE score metric is defined as follows:

$Dice \left( A, B \right)=\frac{2|A \cap B|}{\left| A \right|+|B|}$

The resultant transformation matrix was applied to the source CTA image. This method maximized the registration accuracy within the aorta over other anatomical regions. Minimal variation in the orientation of the bowel/air bubbles was observed between the contrast-enhanced and non-contrast slices. Of note, the registration accuracy for all patients was 95.3 ± 1.2% as assessed by the DICE score.

***Extraction of aortic structure, 2D-Sub-Sampling and Spatial normalization of NCCT/CTA Image Volumes***

The segmented and registered volumes were used to extract the aortic structure within the entire CT image series of each patient. This was done to focus the subsequent deep learning architectures on the aorta/aneurysm and not on surrounding structures. Additionally, the 3D-isolated aorta from both NCCT and CTA images was divided into 2D axial slices. Subsequently, the aorta in each axial slice (512 x 512) was repositioned to the center of that slice (at [256,256]). This isolation, 2D-extraction and spatial-normalization was fully-automated and was performed to each pair of images to generate the dataset for training and testing.

***Model Architectures: Cycle-GAN and Conditional GAN***

The generator and discriminator components in the Cycle-GAN model architecture (**Fig. S2a**) were explicitly defined as least-squares GAN and a 70 x 70 pixel PatchGAN, respectively. The former incorporates an additional least-squares loss function for the discriminator, which in turn, improves the training of the generative model. On the other hand, the discriminator goes through the image pairs, in 70 x 70 patches, and is trained to classify whether the image under question is “real” or “fake”. In addition to the cycle-GAN, a Pix2Pix-Conditional-GAN was trained and evaluated. Unlike the cycle-GAN, conditional GANs require paired non-contrast and contrast images. The generator and discriminator components in the conditional GAN model architecture were identical to those used in the Cycle GAN (**Fig. S2b).**

***GAN model training***

The Con-GAN and Cycle-GAN models were trained with a learning rate of 2.0 * 10^-5^ for 200 epochs on 256 x 256 images centered around the aorta. For the Cycle-GAN architecture, four networks (2 generators + 2 discriminators) were trained simultaneously and various loss functions were evaluated at each iteration to document model training. In addition to the loss metrics inherent to the networks, an identity mapping and a cycle consistency loss functions were included to ensure appropriate style transfer and regularization of the generator to allow for image translation, respectively. On the other hand, two networks (1 generator + 1 discriminator) were trained for the Con-GAN architecture. Model weights were saved every 10 epochs and intermediate model predictions were generated from the NCCT images within the training cohort. The generated predictions were independently evaluated against the ground truth CTA images to assess model training.

***Shape Characterization of Abdominal Aortic Aneurysms***

The shape of an aortic aneurysm is described as either fusiform or saccular. The former suggests that the aneurysm dilates on all sides of the aorta and appears uniform in shape. On the other hand, a saccular-shaped aneurysm bulges/dilates on one side (asymmetric). Difference in AAA shape may lead to altered hemodynamic conditions and wall stresses within the vessel and have been shown to lead to different clinical outcomes. In this study, aneurysmal shape was quantified using the non-fusiform index (NFI), which is a 3-D shape index that describes the deviation of the aneurysmal sac from an ideal fusiform shape. Derived from Martufi et al., the NFI is based on luminal surface area (S) to the volume of the aneurysmal sac (V) and is compared against that from an idealized fusiform model^5^.

$$NFI =1 -\left( \frac{V_{fusiform}^{\frac{2}{3}}}{S_{fusiform}} \right)\left( \frac{S}{V^{2/3}} \right)$$

This idealized fusiform aneurysm, initially proposed by Finol et al. (2002), utilizes the patient-specific neck diameter (D_neck_), maximum AAA diameter (D_max_), and height of the aneurysmal sac (H) and is modeled by the following equation:

$$f_{fusiform}\left( z \right)=\left( \frac{D_{max} -D_{neck}}{4} \right)\left[ 1+\sin\left( \frac{2\pi z}{H}-\frac{\pi}{2} \right) \right]+\frac{D_{neck}}{2}$$

In order to investigate the role AAA size and shape on GAN performance, maximum diameter and NFIs of AAAs were correlated against GAN transformation accuracy, as measured by the DICE score accuracy of the inner lumen.

**Supplementary Tables**

| **Ground-Truth**  **vs  Generated CTA** | | **Conditional-GAN** | | | **Cycle-GAN** | | | **Cycle-GAN (expanded Aortic ROI)** | | |
| --- | --- | --- | --- | --- | --- | --- | --- | --- | --- | --- |
|  |  | **Excluded (n=35)** | **Included (n=165)** | **P**  **value** | **Excluded (n=35)** | **Included (n=165)** | **P**  **value** | **Excluded (n=35)** | **Included (n=165)** | **P value** |
| **DICE (%)** | | 70.0 ± 18.3 % | 83.2 ±  7.7 % | <0.001 | 70.4 ± 20.9 % | 84.1 ±  7.2 % | <0.001 | 78.8 ± 14.9 % | 84.6 ± 9.0 % | **0.01** |
| **Root Mean Square Error (RMSE)** | **Pixel by Pixel comparison** | 4.5 ± 3.8 | 4.2 ± 3.7 | 0.41 | 4.9 ± 4.2 | 4.2 ± 3.8 | 0.32 | 4.3 ± 2.5 | 4.1 ± 2.3 | 0.19 |
|  | **Max Lumen Diameter (mm)** | 12.4 ± 8.5 | 5.2 ± 6.2 | <0.001 | 10.4 ± 7.5 | 4.6 ± 6.7 | <0.001 | 9.9 ± 10.1 | 4.6 ± 5.5 | **<0.01** |
|  | **Max Outer Diameter (mm)** | 1.8 ± 1.7 | 1.5 ± 1.6 | 0.46 | 1.2 ± 1.6 | 1.1 ± 1.7 | 0.56 | 1.5 ± 1.2 | 1.2 ± 1.0 | 0.46 |
| **%-Difference** | **Lumen Area** | 51.2 ± 24.6 % | 25.1 ± 15.2 % | <0.001 | 41.2 ± 33.9 % | 24.1 ± 13.8 % | <0.001 | 46.2 ± 35.9 % | 22.1 ± 15.9 % | **<0.001** |
|  | **Lumen Volume** | 45.6 ± 29.9 % | 22.1 ± 18.6 % | <0.001 | 33.3 ± 26.9 % | 19.1 ± 17.8 % | <0.001 | 34.3 ± 24.6 % | 17.1 ± 15.8 % | **<0.001** |
|  | **ILT Volume** | 36.2 ± 22.2 % | 26.2 ± 24.0 % | <0.001 | 26.0 ± 28.2 % | 22.4 ± 23.4 % | <0.001 | 28.0 ± 30.2 % | 19.9 ± 16.9 % | **<0.001** |

**B.**

**C.**

**A.**

**Table S1:** Ground truth CTA images vs. generated CTA images of the validation cohort. Of the 200 cases, 35 met the exclusion criteria (tube current < 80 mA) as described in the manuscript.

| **Total**  (n = 165) | **Celiac  Artery** | **Sup. Mesenteric Artery** | **Left Renal Artery** | **Right Renal Artery** | **Left Iliac Artery** | **Right Iliac Artery** |
| --- | --- | --- | --- | --- | --- | --- |
| **Branch Capture Rate** | 163/165 | 164/165 | 159/165 | 161/165 | 163/165 | 162/165 |
| **CTA** | 8.2 ± 1.3 mm | 8.5 ± 1.5 mm | 6.8 ± 1.3 mm | 6.7 ± 1.3 mm | 14.9 ± 2.6 mm | 14.8 ± 2.4 mm |
| **Pseudo-Contrast** | 8.3 ± 1.2 mm | 8.4 ± 1.2 mm | 6.7 ± 1.1 mm | 6.6 ± 1.0 mm | 15.0 ± 2.5 mm | 15.1 ± 2.5 mm |
| **MAE** | 0.8 ± 0.5 mm | 0.9 ± 0.5 mm | 0.7 ± 0.6 mm | 0.7 ± 0.5 mm | 0.8 ± 0.5 mm | 0.9 ± 0.4 mm |
| **P** | 0.69 | 0.21 | 0.36 | 0.31 | 0.52 | 0.56 |

**Table S2:** Diameter measurements of aortic side branches from CTA and Pseudo-contrast CT images.

**Supplementary Figures**

**
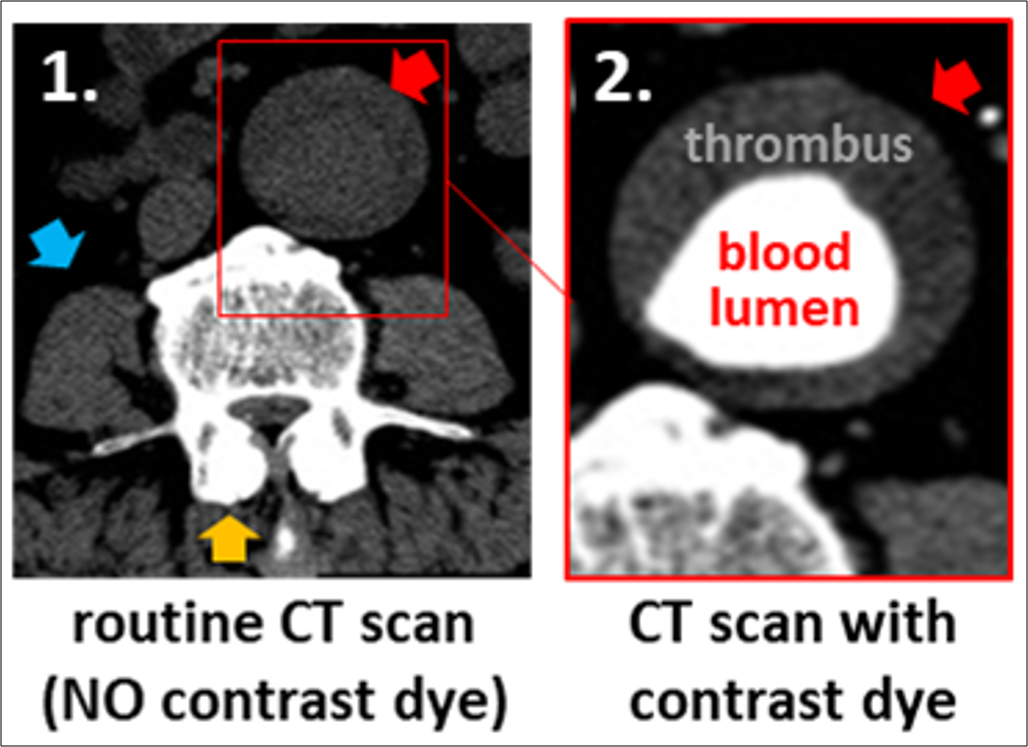
**

**Fig. S1. Axial slice from a Computed Tomography (CT) scan with and without the use of an intravenous iodinated contrast agent.** IV contrast enhances visualization of the vasculature and allows for diagnosis of vascular pathology.


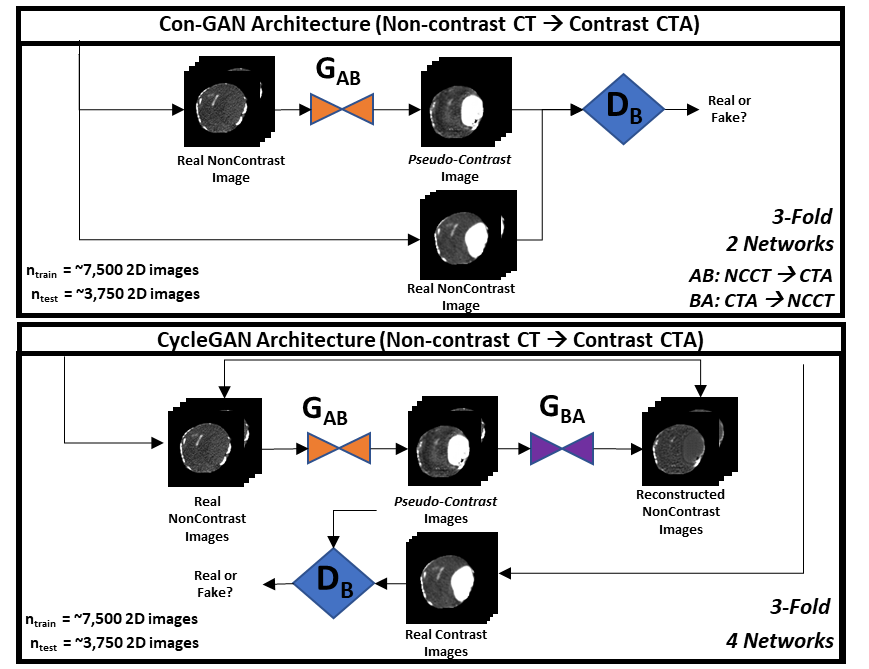


**A.**

**B.**

**Fig. S2. Con-GAN (A.) and CycleGAN (B.) architectures used for the transformation of NCCT images to contrast CTA images**. The former consists of 2 networks (1 generator and 1 discriminator) and the latter consists of 4 networks (2 generators, and 2 discriminators). Each model was trained using a 3-fold cross-validation paradigm.


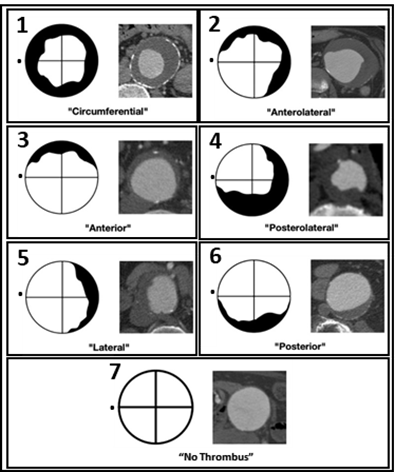


**Fig. S3. Regional classification of thrombus morphology.** Thrombus presence is classified into seven distinct categories (1-7). This is adopted from our previous work^6^.

**
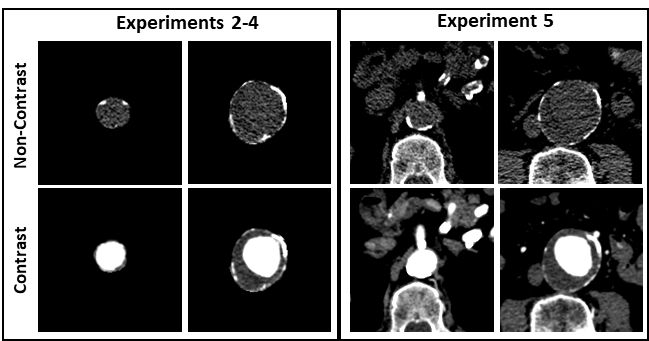
**

**Fig. S4. Input data for the GAN architectures. A.** For experiments 2-4 (Cycle-GAN vs Con-GAN), the input data consisted of 240*240 mm images (256 x 256 pixels) centered around the aorta with all surrounding structures removed. This allowed for the GAN to only focus on the AAA transformation. **B.** For experiment 5, the input data consisted of 135*135 mm (144 x 144 pixels) images centered around the aorta. Extra-aortic structures including side-branches were not removed. This allowed the GAN to focus also on the transformation of adjacent structures.


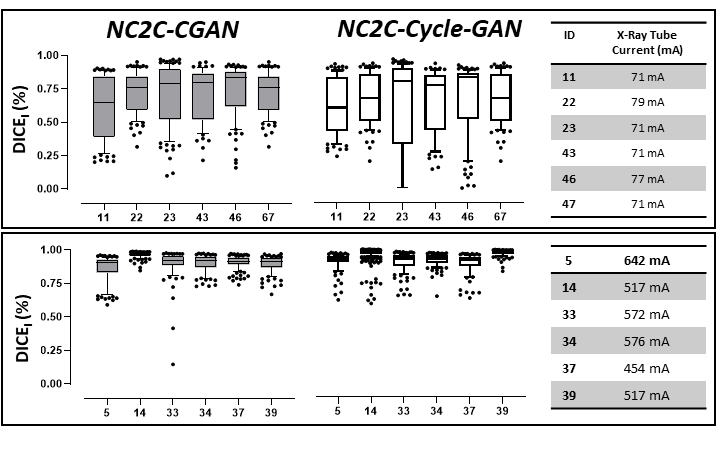


**Fig. S5**. **Increased DICE score variability in NCCT images obtained with low X-Ray tube currents (mA).** Patients (n =6) with the lowest reconstruction accuracy **(A**, Tube Current: 73.3 ± 3.67 mA**)** are compared against those (n = 6) with the highest reconstruction accuracy **(B**, Tube Current: 546.3 ± 64.7 mA**).** X-ray tube currents for the NCCT images are shown accordingly.

**
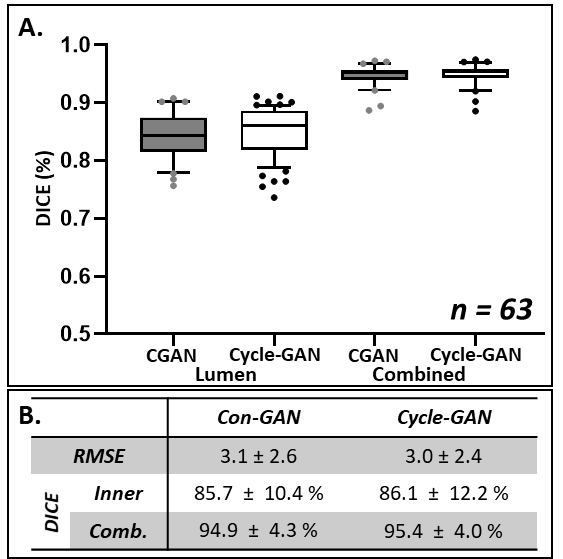
**

**Fig. S6. Transformation accuracy of the refined cohort. A.** Box Plots of averaged DICE scores per patient within the refined cohort (I_tube_ > 80 mA, n = 63) for the lumen (DICE_I_) and the combined aortic mask (DICE_C_) segmentations. **B.** Overall RMSE and DICE scores of pseudo-contrast images from NCCT images obtained at >80 mA.


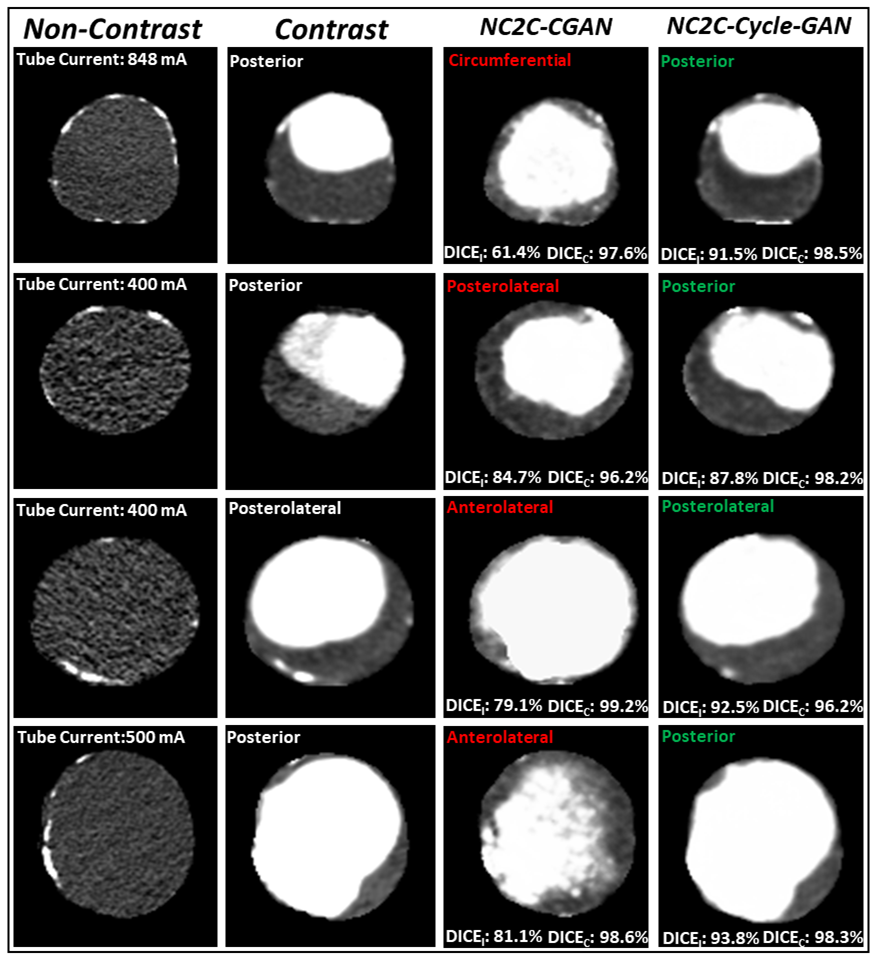


**Fig. S7.** **Points of discrepancy between the NC2C-CGAN and NC2C-Cycle-GAN Models.** Axial images of four patients within the testing cohort are displayed where the NC2C-CGAN fails to properly classify the ILT. However, the NC2C-Cycle-GAN is able to properly classify the ILT. Corresponding x-ray tube currents and DICE scores are highlighted within each panel.

**
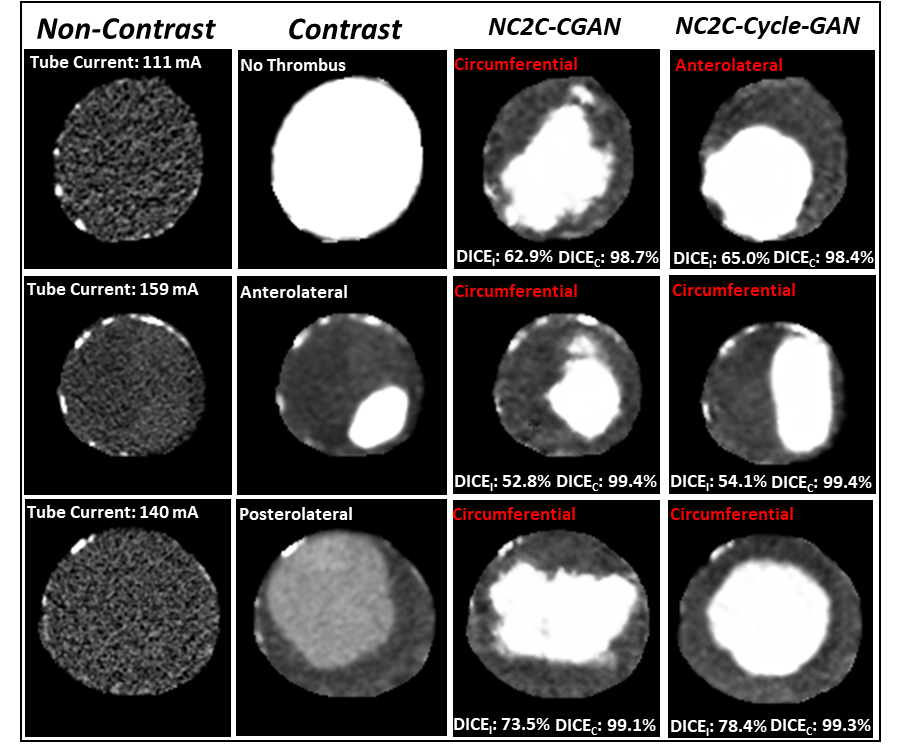
Fig. S8.** **Failure of both generative models.** Axial images of three patients within the testing cohort are displayed where both models fail to properly classify the ILT. Corresponding tube currents and DICE scores are highlighted within each panel.


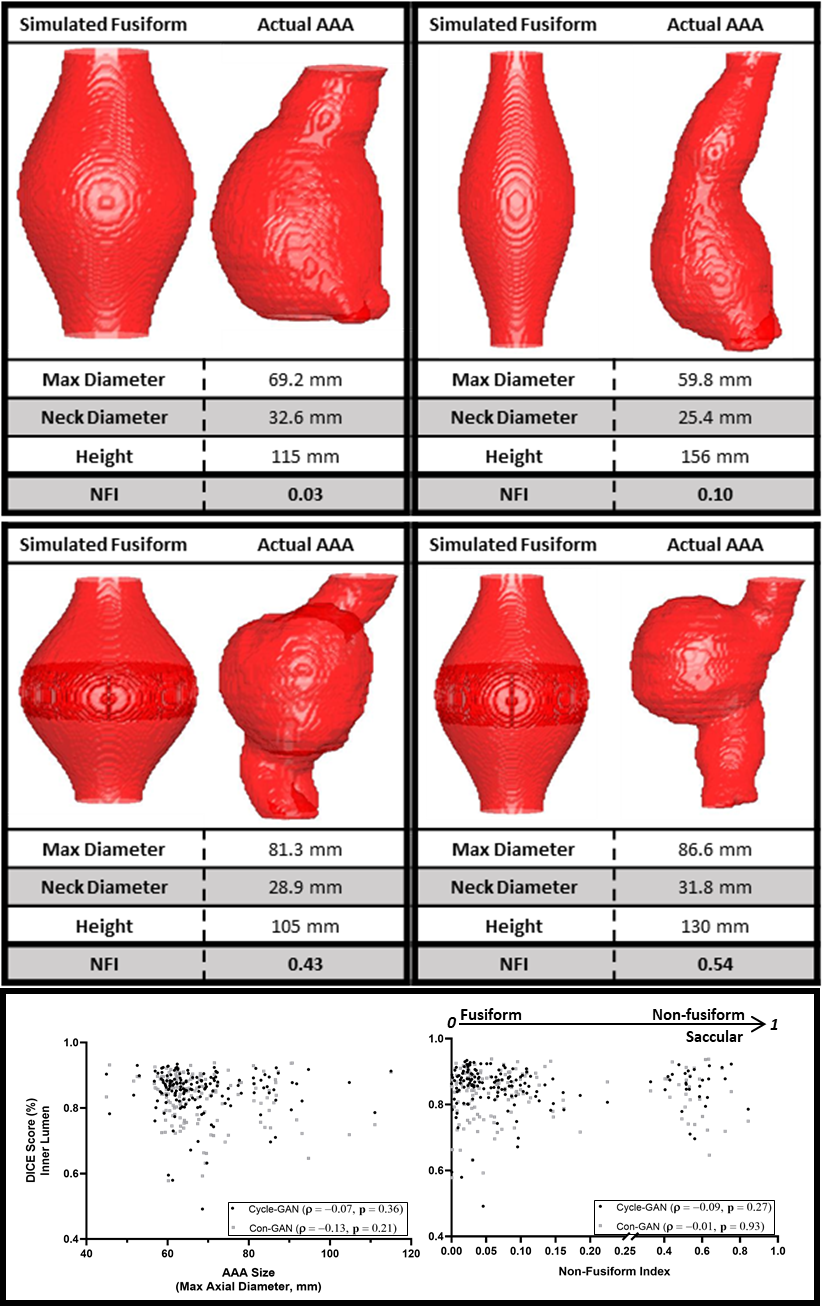


**D.**

**C.**

**B.**

**A.**

**E.**

**Fig. S9: Influence of AAA Size and Shape on GAN Performance.** **A-D**: Four abdominal aortic aneurysms from the validation cohort alongside their respective idealized fusiform models, generated using patient-specific AAA maximum/neck diameters and height of aneurysmal sac. NFIs, a surrogate measure of AAA shape, are displayed for the 4 patients. **E.** Impact of AAA size (Max Diameter) and shape (NFI) on Cycle/Con-GAN transformation accuracy (DICE score of Inner Lumen).


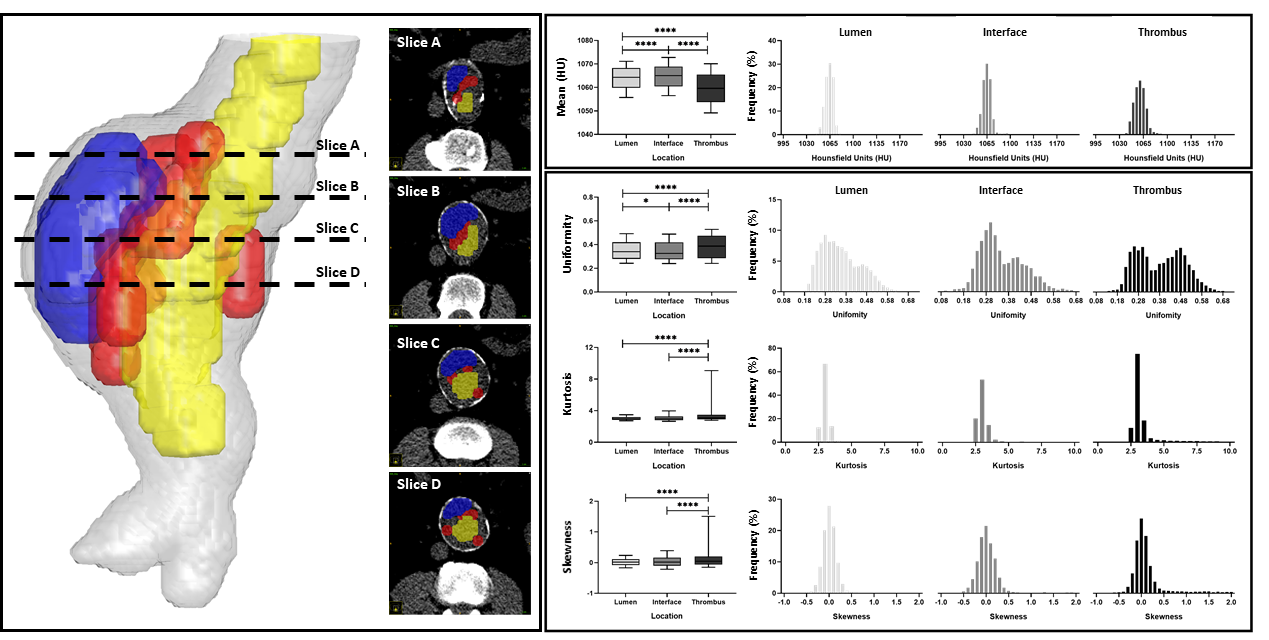


**A.**

**B.**

**Fig. S10.** **A.** Regional sub-sampling (Blue – Thrombus, Red – Interface, Yellow – Lumen) of the aneurysmal sac in NCCT images. This regional sampling method is similar to that presented in Fig 1a. Sub-volumes (1cm x 1cm 1 cm) within each region and without overlap into adjacent regions were sampled from the segmentations (n = 200 patients) resulting in a total of 49,040 sub-volumes (Lumen: 33,278, Interface: 2621, Thrombus: 13,141). Radiomic feature extraction and analysis from these isotropic sub-volumes was performed using Python.  **B.** Extraction of multiple first-order radiomic features (ex. uniformity, kurtosis and skewness) from these distinct regions suggests there are statistically significant differences in addition to that observed with the mean HU intensity. These differences may provide mechanistic insight into the DL generative models and support the validity of the generated images.

1 Chandrashekar, A. *et al.* A Deep Learning Pipeline to Automate High-Resolution Arterial Segmentation with or without Intravenous Contrast. *Ann Surg*, doi:10.1097/SLA.0000000000004595 (2020).

2 Chandrashekar, A. *et al.* A Deep Learning Approach to Automate High-Resolution Blood Vessel Reconstruction on Computerized Tomography Images With or Without the Use of Contrast Agent. (2020).

3 Yushkevich, P. A. *et al.* User-guided 3D active contour segmentation of anatomical structures: significantly improved efficiency and reliability. *NeuroImage* **31**, 1116-1128, doi:10.1016/j.neuroimage.2006.01.015 (2006).

4 Rueckert, D. *et al.* Nonrigid registration using free-form deformations: application to breast MR images. *IEEE Trans Med Imaging* **18**, 712-721, doi:10.1109/42.796284 (1999).

5 Martufi, G., Di Martino, E. S., Amon, C. H., Muluk, S. C. & Finol, E. A. Three-dimensional geometrical characterization of abdominal aortic aneurysms: image-based wall thickness distribution. *J Biomech Eng* **131**, 061015, doi:10.1115/1.3127256 (2009).

6 Whaley, Z. L. *et al.* The Spatial Morphology of Intraluminal Thrombus Influences Type II Endoleak after Endovascular Repair of Abdominal Aortic Aneurysms. *Ann Vasc Surg*, doi:10.1016/j.avsg.2019.05.050 (2019).
